# Mint for the Suppression of the Milk

**Published:** 1874-04

**Authors:** 


The Employment of Mint for the Suppression of the Milk.—Dr. Dasara observes that the knowledge of the anti-lactiferous properties of mint appears to have been possessed in very ancient times, since Dioscorides mentions the fact in his works, and subsequent writers have only confirmed his statement.

1. It is an established fact that mint has the power of suppressing the lacteal secretion. 2. The suppression of the secretion takes place at whatever period of lactation the mint is employed. 3. The effect takes place in a very short space of time; according to his experiments, in from three to five days. 4. The suppressive action of mint can be localized to one breast. 5. No danger, nor even any inconvenience, arises, either to the mother or child, either from the use of the mint or from the suppression of the secretion.

